# Aortic aneurysm and aortic graft infection related to *Mycobacterium bovis* after intravesical Bacille Calmette–Guérin therapy—a case series

**DOI:** 10.1186/s12893-021-01142-1

**Published:** 2021-03-17

**Authors:** M. Buerger, S. Kapahnke, S. Omran, M. Schomaker, M. Rief, A. Greiner, J. P. Frese

**Affiliations:** 1 Department of Vascular and Endovascular Surgery, Charité – Universitätsmedizin Berlin, corporate Member of Freie Universität Berlin and Humboldt-Universität zu Berlin, Hindenburgdamm 30, 12203 Berlin, Germany; 2 Institute of Radiology, Charité – Universitätsmedizin Berlin, corporate member of Freie Universität Berlin and Humboldt-Universität zu Berlin, Luisenstraße 10, 10117 Berlin, Germany

**Keywords:** Case report, Aortic surgery, Mycotic aortic aneurysm, Graft infection, Bladder cancer, *Mycobacterium bovis*

## Abstract

**Background:**

So called “mycotic” aortic aneurysms account for only 0.7 to 1.3% of all aortic aneurysms and are commonly caused by Staphylococcus aureus and Salmonella species. Bacillus Calmette-Guérin (BCG), a live attenuated strain of *Mycobacterium bovis*, is part of the therapy of non-muscle-invasive bladder cancer (NMIBC).

**Case presentation:**

We report a case series of three patients with a mycobacterial graft infection related to BCG after surgical treatment of a presumed mycotic aortic aneurysm as an extremely rare complication after NMIBC treatment. All three patients developed aortic aneurysm after BCG instillation and subsequent mycobacterial graft infection.

**Conclusion:**

Diagnosis requires a high degree of suspicion because of its nonspecific symptoms and imaging. The pathogen is not detected by standard microbiological testing. Treatment includes triple antimycobacterial therapy and radical surgical interventions. Graft preservation may be considered if no anastomosis is involved.

**Supplementary Information:**

The online version contains supplementary material available at 10.1186/s12893-021-01142-1.

## Background

Infectious or so called ‘mycotic’ aortic aneurysms represent merely 0.7 to 1.3% of all aortic aneurysms. Nevertheless, they constitute a life-threatening vascular complication associated with in-hospital mortality rates of up to 36% [1, 2]. Mycotic aortic aneurysms are usually caused by Staphylococcus aureus, followed by Salmonella spp., Streptococcus spp. and Escherichia coli. Mycotic aneurysms caused by mycobacterial infections are rare. Up to now, 44 cases in 42 case reports of mycotic aortic aneurysms caused by mycobacterial infection affecting the thoracoabdominal aorta after intravesical instillation of Bacillus Calmette–Guérin (BCG) have been reported (see Additional file 1: Table S1).

Intravesical BCG applications are standard of care for treating non-muscle-invasive bladder cancer (NMIBC) [3]. The live attenuated strain of *Mycobacterium bovis* is administered to patients with NMIBC after transurethral resection of bladder tumors (TURBT) to avert recurrence and reduce progression rates of intermediate- and high-risk tumors [4]. The incidence of BCG-related systemic complications including spondylodiscitis, psoas abscess or vascular complications remains unclear. In this case series, we report three cases of mycobacterial aortic graft infection related to intravesical application of BCG for NMIBC. This represents the largest case series in the current literature. The particular challenges for the diagnosis and treatment of this infrequent complication are discussed in this report.

## Case presentations

### Patient A

**Fig. 1:**
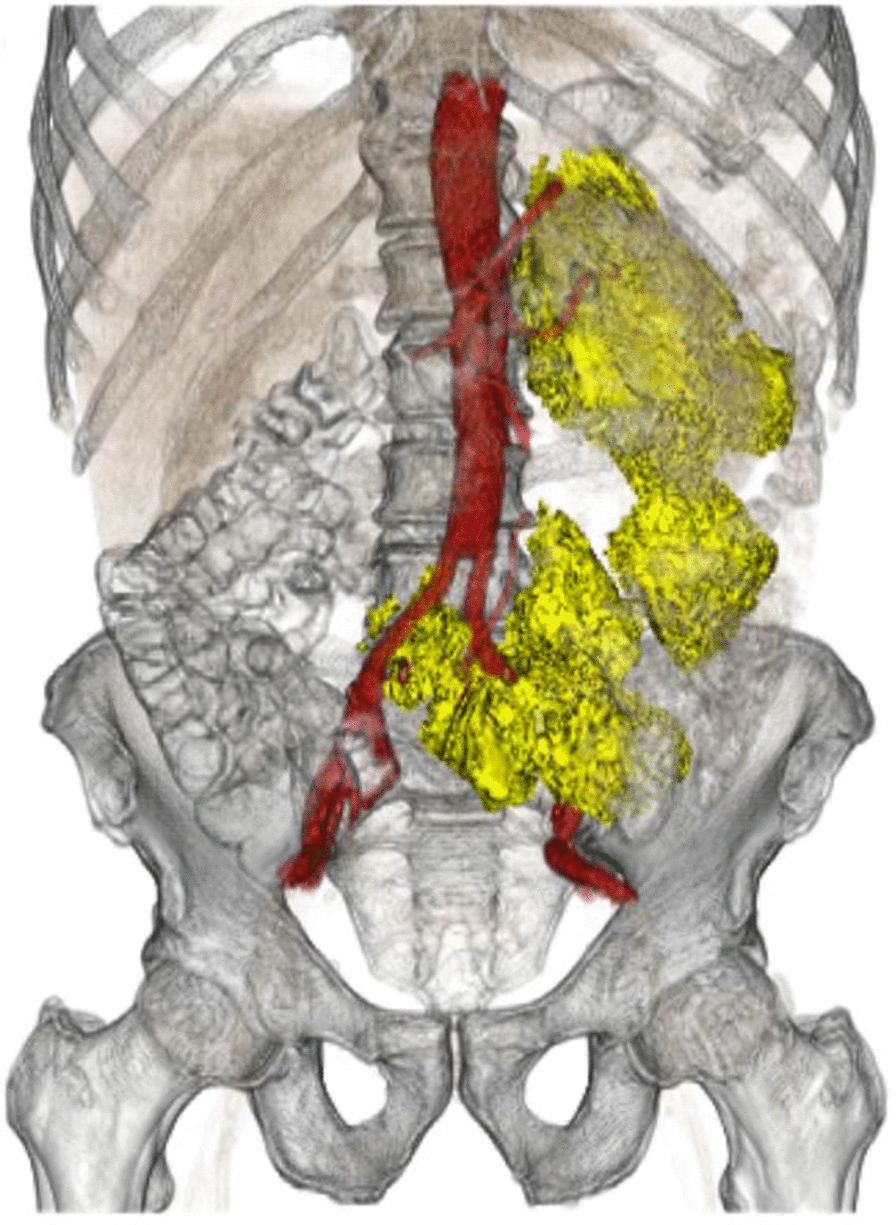
CT- reconstruction showing massive retroperitoneal fluid mass affecting left branch of aortobiiliac graft

In November 2010, a 63-year-old man was referred to our clinic because of persistent weakness, fever, night sweat and unspecific abdominal pain lasting for two weeks. Three months before this episode, the patient had undergone emergency surgery for a ruptured infrarenal abdominal aortic aneurysm, which was treated with implantation of an aortobiiliac graft at another clinic. In 2008, the patient had been treated for NMIBC by transurethral resection and had received three cycles of intravesical BCG instillations (see Table 1). At the time of presentation, clinical examination and blood tests were unremarkable for inflammation except for a temperature of 37.6 °C. Ultrasound revealed a retroperitoneal fluid collection. Computed tomography (CT) demonstrated an extensive low-density collection surrounding the aortic prosthesis. The retroperitoneal mass was surgically removed and tissue samples were collected for histologic and microbiological studies. A specimen of the necrotic mass indicated chronic inflammation but tested negative for acid-fast bacilli (AFB) in Ziehl–Neelsen stain (Artisan™ Acid-Fast Bacillus (AFB) Stain Kit, Agilent, Santa Clara, United States). As no evidence for mycobacterial infection was found, the patient was discharged home in good condition, without antitubercular medication. Another two years later in August 2012, he presented again to our clinic with fever (40.4 °C), elevated white blood cell count (17.92/nl) and increased C-reactive protein (14.4 mg/l). CT scan revealed recurrence of a large mass in the retroperitoneum (Fig. 1). The patient underwent CT-guided aspiration. A drain was placed and specimens were sent to the microbiological laboratory. Microscopic examination showed necrotizing granulomatous inflammation with multinucleated giant cells. Direct Polymerase-chain-reaction (PCR, cobas® 6800 MTB Test, Roche, Rotkreuz, Switzerland) tested positive for Mycobacterium tuberculosis complex. Growth of *Mycobacterium bovis* in solid (BBL Stonebrink TB Medium + PACT + BBL Löwenstein-Jensen; Becton Dickinson, Rungis, France) and liquid culture (MGIT BBL; Becton Dickinson, Rungis, France) confirmed the suspected diagnosis. Antitubercular therapy with Isoniazid (INH), Rifampicin (RFP) and Ethambutol (EB) was started and the fluid collection was removed surgically. Intraoperatively, a partial affection of the left leg of the bifurcated graft by the necrotic mass was detected. After extensive debridement, vacuum assisted wound therapy was initiated. The possibility of an aortic reconstruction using autologous deep femoral vein or cryo-preserved homograft was evaluated but declined by the patient. After four weeks of negative pressure wound therapy, the wound could be closed in secondary intention. CT- scan showed no signs of persistent graft infection or retroperitoneal abscess. The patient was discharged in good condition. The triple antitubercular treatment was continued for six month and reduced to RFH and INH for another six months. As the patient remained free of symptoms, and CT scan was unremarkable, antitubercular therapy was subsequently terminated. After a follow-up of 84 months, the patient is in excellent condition and there is no CT- morphologic sign of recurrence of retroperitoneal abscess or inflammation of vascular graft.

**Table 1:** Patient characteristics

Preliminary diagnoses	Preliminary surgical procedures
Patient A	
NMIBC in 2008	Aortobiiliac prosthesis in 2010
Ruptured aortic aneurysm in 2010	
Chronic obstructive pulmonary disease	
Disc prolaps L5/L6	
Renal cysts	
Patient B	
NMIBC in 2014	None
Disc prolaps L2/L3 and L3/L4	
Patient C	
NMIBC in 2008	Aortocoronary bypass operation in 2011
Coronary artery disease	Inguinal hernia repair
Cardial arrhythmia type II Wenckebach	Dacron tube graft in 2015
Arterial hypertension	
Hyperlipidemia	

*NMIBC* Non-muscle-invasive bladder cancer

### Patient B

**Fig. 2:**
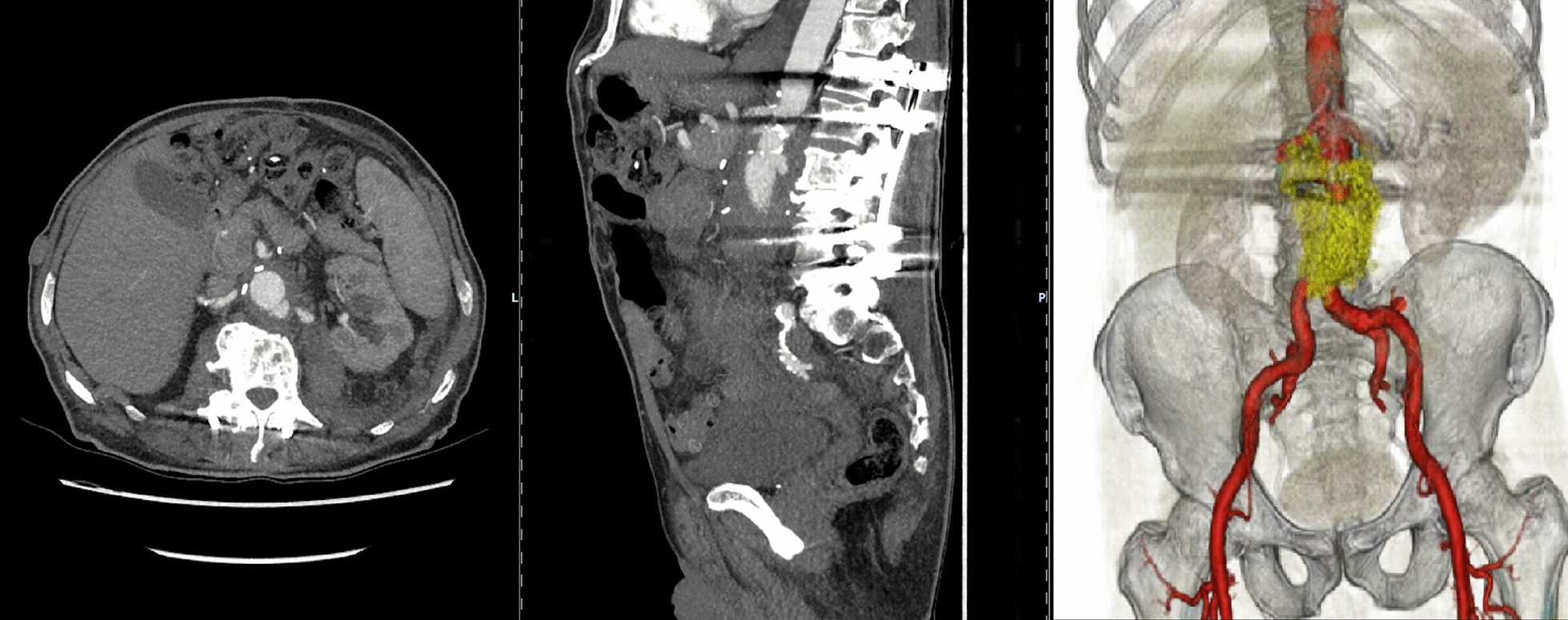
Progress of periprosthetic fluid mass highly suspicious for mycobacterial graft infection and anastomotic pseudoaneurysm of the former implanted Dacron tube graft

In January 2018, a 78-year-old man presented with acute abdominal pain after a six-week history of chronic back pain. CT scan revealed a small-sized saccular aneurysmatic formation suspicious for a penetrating aortic ulcer (PAU) with contained rupture in the infrarenal aorta. Bone lesions in the vertebral bodies L2/L3 were highly suspicious for concomitant spondylodiscitis. The PAU was too small in size and anatomically too remote to represent a likely cause. Retrospectively, CT scan from 2014 did not show an aortic or vertebral pathology. The patient’s medical history was significant for NMIBC treated with TURBT and multiple intravesical BCG applications in 2014 (Table 1). The patient was hemodynamically stable and showed no notable physical examination findings. Except for increased C-reactive protein (25.1 mg/l), laboratory parameters were within normal limits. Because of close proximity of the PAU to the renal arteries resulting in a short neck, endovascular treatment was rejected. Immediate aortic reconstruction was performed by implantation of a rifampicin-soaked Dacron tube graft. Pathological examination and microbiological testing showed no evidence of mycobacterial infection or other pathogens in intraoperative specimens of the infrarenal aorta. The patient had an uneventful recovery and was discharged after two weeks. After four months of persisting lower back pain, biopsy extraction and spondylodesis of the concomitant spondylodiscitis was performed. The microbiological analysis tested negative for AFB in Ziehl–Neelsen stain but the growth of *Mycobacterium bovis* in solid and liquid culture confirmed the diagnosis of mycobacterial infection. Antitubercular treatment consisting of RFP, INH, and EB was administered. One year later, follow-up CT scan revealed dislocation of the former implanted spondylodesis which was surgically removed and replaced. Intraoperative samples showed no evidence of persisting mycobacterial infection with acid fast medium culture and PCR. The patient was discharged after eight days. Antitubercular triple therapy was continued for another six months. During follow-up of 20 months, the patient presented free of symptoms. CT scan showed a shrinking psoas abscess. Three months later, the patient presented to the emergency room with increasing left lower abdominal pain. CT examination showed a false aneurysm near the proximal anastomosis of the Dacron tube graft and a retroperitoneal periprosthetic fluid collection (Fig. 2), indicating an imminent rupture. Graft explantation and replacement with homograft was performed. Intraoperative sample material did not show any evidence of *Mycobacterium bovis* nor other pathogens. Due to the earlier confirmation of BCG- related graft infection, the anti-mycobacterial therapy was continued for another 12 months with Doxycyclin, RFP and INH. After an uncomplicated course, the patient was discharged to a rehabilitation center two weeks after surgery. Follow-up imaging was performed five months later revealing no residual fluid collection. The patient remained in good clinical condition without any signs of re-infection.

### Patient C

**Fig. 3:**
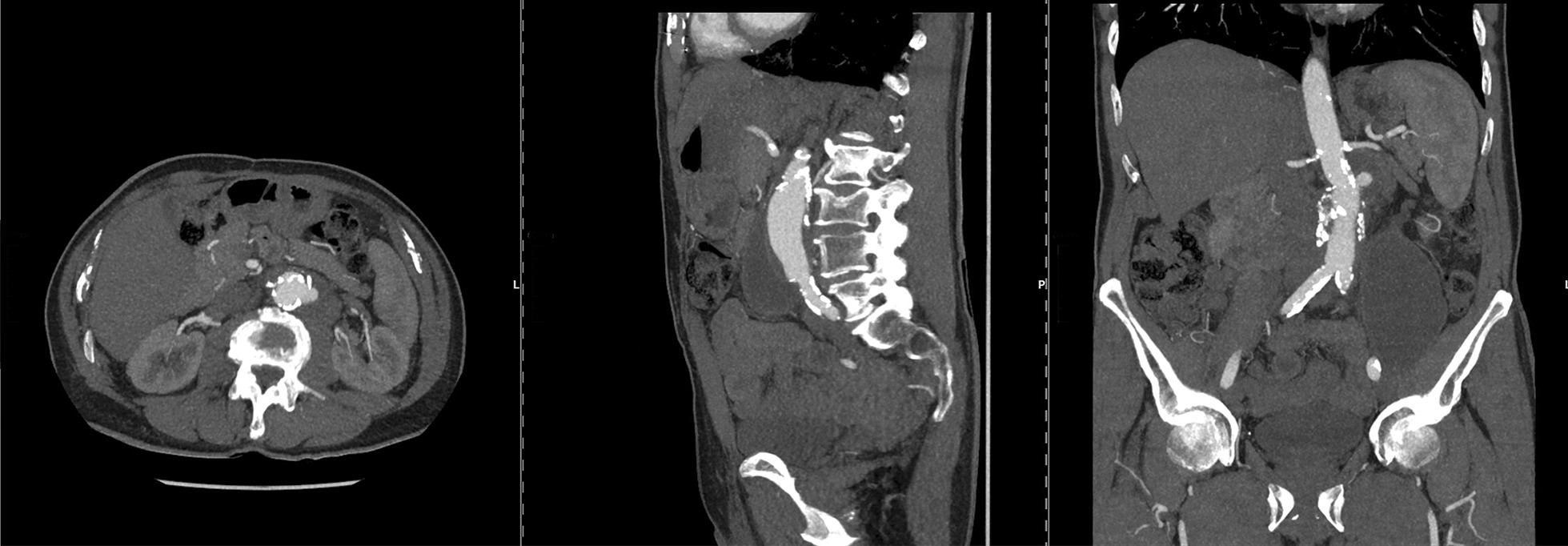
Anastomotic pseudoaneurysm and progression of both periprosthetic fluid mass and fluid collection in left psoas muscle

In December 2017, a 79-year-old man presented as an outpatient to our clinic with night sweats and persisting weakness after treatment of a contained rupture of an infrarenal PAU with implantation of a Dacron tube graft at an external institution in 2015. Ultrasound examination and CT showed periprosthetic fluid collection. The patient’s medical history included NIMBC diagnosed in 2008 and treated with TURBT and a total of 36 intravesical BCG instillations (Table 1). Laboratory parameters were within normal limits (CRP: 4.7 mg/l; white blood cell count: 4.08/nl). Samples collected by CT-guided aspiration tested positive for AFB with Auramine-rhodamine stain (Wescor Aerospray TB 7722, ELITech Biomedical Systems, Logan, Utah, USA). PCR of the aspirated fluid was positive for Mycobacterium tuberculosis complex and incubation in solid and liquid culture showed growth of *Mycobacterium bovis*. Antimycobacterial treatment was initiated. Because of a critical interaction between Ranolexin (long-term medication), Rifampicin was replaced by Rifabutin in combination with INH and EB. After a follow-up period of six months, control CT scan revealed a false aneurysm in the infrarenal region near the proximal anastomosis of the Dacron tube graft and a progression of the fluid collection in the psoas muscle (Fig. 3). The Dacron tube graft was completely removed and replaced by deep femoral vein (Fig. S1 in Additional file 2). Despite a weak positive PCR for Mycobacterium complex, culture incubation showed no growth of *Mycobacterium bovis*. This was considered to be the result of an effective perioperative antimycotic therapy. After an uncomplicated postoperative course, the patient was discharged to an external rehabilitation center after 25 days. During routine outpatient follow-up 12 months after surgery, the patient was in good general condition. The CT scan showed no persistent prosthetic infection.

## Discussion and conclusion

Intravesical instillation of BCG is widely used for the treatment of NMIBC. First used as a vaccine against tuberculosis, intravesical instillation of the live attenuated strain of *Mycobacterium bovis* has become standard care for NMIBC. The exact mechanism of its antitumor efficacy is not clarified yet. Although BCG instillation is generally considered safe, severe side effects have been reported. The incidence of mycotic aortic aneurysms caused by disseminated tuberculosis after BCG instillation remains unclear. Three different mechanisms are described that could lead to a systemic dissemination of and vascular affection by *Mycobacterium bovis*: (1) hematogenous dissemination followed by direct intimal colonization favored by preexisting atherosclerosis, (2) metastatic implantation through the vasa vasorum, or (3) continuous dissemination of adjacent infected tissue such as contiguous lymphadenitis or psoas abscess [5].

We report three cases of graft infection after surgical treatment of suspected mycotic aortic aneurysms as an extremely rare complication after intravesical BCG application. Our therapy was based on two pillars: drug-based antitubercular therapy as well as the individually adapted surgical treatment consisting of radical surgical debridement, vacuum therapy, drainage and in case of anastomotic leakage, graft explantation and aortic reconstruction using deep femoral vein or homograft.

So far, 44 cases of mycotic aortic aneurysms caused by disseminated mycobacterial infection have been published. Foreign material was implanted in 28 patients [6–32]. Postoperative graft infection was diagnosed in 10 out of 28 cases (35.7%) [6, 12, 17, 19–22, 32–34]. Of these 10 patients, six were discharged resulting in a hospital survival rate of 60%. A long-term follow-up of more than 12 months is reported by only one author [33]. Treatment included medical therapy alone (n = 1) [6], invasive therapy including either CT-guided drainage or surgical debridement with graft preservation (n = 2) [12, 19] and radical surgical therapy with partial (n = 1) [17] or complete graft explantation (n = 6) [20–22, 32–34]. Antitubercular medication was administered in 8 out of 10 cases [6, 12, 17, 19–22, 33]. Apart from one case [33], graft explantation was performed combined with aortic stump ligation and implantation of an extraanatomic axillofemoral bypass. Similar to our patients B and C, Santbergen et al. (33) focused on aortic reconstruction with autologous replacement material with an excellent postoperative result. Eighteen months after completion of the antimycobacterial medication, there was no clinical and morphological evidence of a persistent infection.

Diagnosis of mycotic aneurysms or vascular complications after intravesical BCG application is exceptionally challenging and a high level of suspicion is required. A thorough medical history with attention to past NMIBC and related BCG instillations is essential when a mycotic aneurysm or graft infection is suspected. Unspecific symptoms like night sweats, weight loss, malaise and persisting fever combined with an unremarkable clinical examination can draw suspicion to a chronic disease like disseminated BCG- infection. After obtaining sample material from affected regions, e.g. vertebral disc, fluid collection in psoas muscle, aortic wall, paraaortic lymph nodes, pathological and microbiological examination is required. Suspected diagnosis can only be confirmed by PCR and culture specific for mycobacterial pathogens. Standard microbiological testing is often negative.

All of our three patients had received BCG therapy several years before the primary intervention. Because of the slow replication rate of mycobacteria, symptomatic vascular disease will often occur with a long latency after BCG instillation. Delay in diagnosis will lead to a delay in starting antimycobacterial treatment, which may result in an increased risk of rupture and dissemination, further limiting surgical options.

Currently, no consensus guideline for optimal medical treatment options of graft infection secondary to BCG instillation is available. Given its rareness, a case by case decision has to be made. Antitubercular therapy should be administered immediately after confirmed diagnosis and should be continued for at least six to 12 months. This medical therapy should be supplemented by surgical treatment. Our first case was managed successfully without graft explantation, with repeated radical surgical debridement and vacuum therapy alone, as no anastomosis was involved in this case. In our two following cases, anastomotic aneurysm indicating imminent rupture was discovered during follow-up. In these cases, we decided to completely remove the infected graft and to reconstruct the aorta with deep femoral vein or homograft, respectively.

In conclusion, this three-case series represents our experience with this rare condition. We strongly suggest that in cases of aortic aneurysm or aortic graft infection in patients with previous BCG instillation, surgeons should be suspicious of BCG- infection and mycotic aneurysm and to initiate specific testing including PCR and culture for mycobacterial infection. In cases with involvement of any graft anastomosis, complete graft removal and replacement with biological material should be considered. In cases with no involvement of an anastomosis, graft preservation could be attempted. Adjuvant antimycobacterial treatment is essential and should be initiated as soon as diagnosis is confirmed.

## Supplementary Information

**Additional file 1: Table S1.** Publications reporting aortic aneurysm secondary to intravesical application of Bacillus Calmette Guérin

**Additional file 2: Fig. S1.** Intraoperative situs showing exit of purulent granular material from the aneurysmatic formation (A) surrounding former implanted Dacron tube graft (B) and aortic reconstruction after graft removal and reconstruction with deep femoral vein (C)

## Data Availability

The data collection was done by reviewing the available literature and electronic health records at the Charité Universitätsmedizin Berlin. Relevant data was collected in Microsoft Excel and analysed descriptively. The data is stored on the Charité—server and, in order to avoid a violation of access rights, the data is encrypted using a password only known to the study physicians. The data that support the findings of this study is available, but restrictions apply to the availability of the data, which was used under license for the current study, and so is not publicly available. Data is however available from the authors upon reasonable request.

## Abbreviations

BCG: Bacille Calmette–Guérin
NMIBC: Non-muscle-invasive bladder cancer
TURBT: Transurethral resection of bladder tumors
CT: Computed tomography
AFB: Acid-fast bacilli
PCR: Polymerase-chain-reaction
PZA: Pyrazinamide
INH: Isoniazid
RFP: Rifampicin
EB: Ethambutol
PAU: Penetrating aortic ulcer
MRI: Magnetic resonance imaging

## References

[1] Muller BT, Wegener OR, Grabitz K, Pillny M, Thomas L, Sandmann W (2001). Mycotic aneurysms of the thoracic and abdominal aorta and iliac arteries: experience with anatomic and extra-anatomic repair in 33 cases. J Vasc Surg.

[2] Oderich GS, Panneton JM, Bower TC, Cherry KJ, Rowland CM, Noel AA (2001). Infected aortic aneurysms: aggressive presentation, complicated early outcome, but durable results. J Vasc Surg.

[3] Morales A, Eidinger D, Bruce AW (1976). Intracavitary Bacillus Calmette–Guerin in the treatment of superficial bladder tumors. J Urol.

[4] Babjuk M, Bohle A, Burger M, Capoun O, Cohen D, Comperat EM (2017). EAU guidelines on non-muscle-invasive urothelial carcinoma of the bladder: update 2016. Eur Urol.

[5] Long R, Guzman R, Greenberg H, Safneck J, Hershfield E (1999). Tuberculous mycotic aneurysm of the aorta: review of published medical and surgical experience. Chest.

[6] Higashi Y, Nakamura S, Kidani K, Matumoto K, Kawago K, Isobe J (2018). *Mycobacterium bovis*-induced aneurysm after intravesical Bacillus Calmette–Guerin therapy: a case study and literature review. Intern Med.

[7] Wadhwani A, Moore RD, Bakshi D, Mirakhur A (2018). Mycotic aortic aneurysms post-Intravesical BCG treatment for early-stage bladder carcinoma. CVIR Endovasc.

[8] Coddington ND, Sandberg JK, Yang C, Sehn JK, Kim EH, Strope SA (2017). Mycotic aneurysm after Bacillus Calmette–Guerin treatment: case report and review of the literature. Case Rep Urol.

[9] Holmes BJ, LaRue RW, Black JH, Dionne K, Parrish NM, Melia MT (2014). Mycotic aortic aneurysm due to intravesical BCG immunotherapy: clinical manifestations and diagnostic challenges. Int J Mycobacteriol.

[10] Floros N, Meletiadis K, Kusenack U, Zirngibl H, Kamper L, Haage P (2015). Ruptured Mycotic Aortic Aneurysm after Bacille Calmette-Guerin Therapy. Ann Vasc Surg.

[11] Davis FM, Miller DJ, Newton D, Arya S, Escobar GA (2015). Successful treatment of a mycotic multifocal thoracoabdominal aortic aneurysm as a late sequelae of intravesical bacillus Calmette–Guerin therapy: case report and literature review. Ann Vasc Surg.

[12] Leo E, Molinari AL, Rossi G, Ferrari SA, Terzi A, Lorenzi G (2015). Mycotic abdominal aortic aneurysm after adjuvant therapy with bacillus Calmette–Guerin in patients with urothelial bladder cancer: a rare but misinterpreted complication. Ann Vasc Surg.

[13] Seastedt KP, Ahmad U, Lau C, Ruggeri-Weigel P, Tsang HC, Hartman BJ (2015). Mycotic thoracic aortic aneurysm after intravesical Bacillus Calmette–Guerin treatment. Ann Thorac Surg.

[14] Akita H, Okamura T, Nakane A, Kobayashi T, Yamada K, Tanaka Y (2015). Infectious aortic aneurysms occurring 1 year after bacillus Calmette–Guerin bladder instillation therapy. Int J Urol.

[15] Nam EY, Na SH, Kim SY, Yoon D, Kim CJ, Park KU (2015). Infected Aortic Aneurysm caused by *Mycobacterium bovis* after Intravesical Bacillus Calmette–Guerin treatment for bladder cancer. Infect Chemother.

[16] Roylance A, Mosley J, Jameel M, Sylvan A, Walker V (2013). Aorto-enteric fistula development secondary to mycotic abdominal aortic aneurysm following intravesical bacillus Calmette–Guerin (BCG) treatment for transitional cell carcinoma of the bladder. Int J Surg Case Rep.

[17] Mizoguchi H, Iida O, Dohi T, Tomoda K, Kimura H, Inoue K (2013). Abdominal aortic aneurysmal and endovascular device infection with iliopsoas abscess caused by *Mycobacterium bovis* as a complication of intravesical bacillus Calmette–Guerin therapy. Ann Vasc Surg.

[18] Harding GE, Lawlor DK (2007). Ruptured mycotic abdominal aortic aneurysm secondary to *Mycobacterium bovis* after intravesical treatment with bacillus Calmette-Guerin. J Vasc Surg.

[19] Roeke T, Hovsibian S, Schlejen PM, Dinant S, Koster T, Waasdorp EJ (2018). A mycotic aneurysm of the abdominal aorta caused by *Mycobacterium bovis* after intravesical instillation with bacillus Calmette–Guerin. J Vasc Surg Cases Innov Tech.

[20] Costiniuk CT, Sharapov AA, Rose GW, Veinot JP, Desjardins M, Brandys TM (2010). *Mycobacterium bovis* abdominal aortic and femoral artery aneurysms following intravesical bacillus Calmette–Guerin therapy for bladder cancer. Cardiovasc Pathol.

[21] Wolf YG, Wolf DG, Higginbottom PA, Dilley RB (1995). Infection of a ruptured aortic aneurysm and an aortic graft with bacille Calmette-Guerin after intravesical administration for bladder cancer. J Vasc Surg.

[22] Lareyre F, Reverso-Meinietti J, Carboni J, Gaudart A, Hassen-Khodja R, Raffort JM (2019). Mycotic aortic aneurysm and infected aortic graft after intravesical Bacillus Calmette–Guerin treatment for bladder cancer. Vasc Endovascular Surg.

[23] Dahl T, Lange C, Odegard A, Bergh K, Osen SS, Myhre HO (2005). Ruptured abdominal aortic aneurysm secondary to tuberculous spondylitis. Int Angiol.

[24] Rozenblit A, Wasserman E, Marin ML, Veith FJ, Cynamon J, Rozenblit G (1996). Infected aortic aneurysm and vertebral osteomyelitis after intravesical bacillus Calmette–Guerin therapy. AJR Am J Roentgenol.

[25] Woods JM, Schellack J, Stewart MT, Murray DR, Schwartzman SW (1988). Mycotic abdominal aortic aneurysm induced by immunotherapy with bacille Calmette–Guerin vaccine for malignancy. J Vasc Surg..

[26] Wada S, Watanabe Y, Shiono N, Masuhara H, Hamada S, Ozawa T (2003). Tuberculous abdominal aortic pseudoaneurysm penetrating the left psoas muscle after BCG therapy for bladder cancer. Cardiovasc Surg.

[27] Damm O, Briheim G, Hagstrom T, Jonsson B, Skau T (1998). Ruptured mycotic aneurysm of the abdominal aorta: a serious complication of intravesical instillation bacillus Calmette–Guerin therapy. J Urol.

[28] Hellinger WC, Oldenburg WA, Alvarez S (1995). Vascular and other serious infections with *Mycobacterium bovis* after bacillus of Calmette–Guerin therapy for bladder cancer. South Med J.

[29] Kusakabe T, Endo K, Nakamura I, Suzuki H, Nishimura H, Fukushima S (2018). Bacille Calmette–Guerin (BCG) spondylitis with adjacent mycotic aortic aneurysm after intravesical BCG therapy: a case report and literature review. BMC Infect Dis.

[30] Seelig MH, Oldenburg WA, Klingler PJ, Blute ML, Pairolero PC (1999). Mycotic vascular infections of large arteries with *Mycobacterium bovis* after intravesical bacillus Calmette–Guerin therapy: case report. J Vasc Surg.

[31] Smith DM (2016). BCG-osis following intravesical BCG treatment leading to miliary pulmonary nodules, penile granulomas and a mycotic aortic aneurysm. BMJ Case Rep..

[32] Berchiolli R, Mocellin DM, Marconi M, Tomei F, Bargellini I, Zanca R (2019). Ruptured mycotic aneurysm after intravesical instillation for bladder tumor. Ann Vasc Surg..

[33] Santbergen B, Vriens PH, de Lange WC, Van Kasteren ME (2013). Combined infection of vertebroplasty and aortic graft after intravesical BCG treatment. BMJ Case Rep..

[34] LaBerge JM, Kerlan RK, Reilly LM, Chuter TA (1999). Diagnosis please. Case 9: mycotic pseudoaneurysm of the abdominal aorta in association with mycobacterial psoas abscess–a complication of BCG therapy. Radiology..

