# Supplementary material for: Aortic aneurysm and aortic graft infection related to Mycobacterium bovis after intravesical Bacille Calmette–Guérin therapy—a case series
Source: BMC Surg. 2021 Mar 17;21:138. doi: 10.1186/s12893-021-01142-1 (PMC7972206; doi:10.1186/s12893-021-01142-1)
Supplement: Supplementary file 1 — Additional file 1: Table S1. Publications reporting aortic aneurysm secondary to intravesical application of Bacillus Calmette Guérin [file 12893_2021_1142_MOESM1_ESM.docx]

Online supplemental table 1

Publications reporting aortic aneurysm secondary to intravesical application of Bacillus Calmette Guérin

| **Author** | **Year of publication** | **Number of reported cases** | **Title** | **Journal** |
| --- | --- | --- | --- | --- |
| *Okon et al. (1)* | 2017 | 1 | Tuberculous Psoas Abscess and Worsening Vascular Aneurysm; All from Bacillus Calmette-Guerin (BCG) Therapy? | The American journal of case reports |
| *Simar et al. (2)* | 2017 | 1 | Ruptured aortic aneurysm due to Mycobacterium bovis BCG with a delayed bacteriological diagnosis due to false negative result of the MPB 64 immunochromatographic assay | BMC research notes |
| *Higashi et al. (3)* | 2018 | 1 | Mycobacterium bovis-induced Aneurysm after Intravesical Bacillus Calmette-Guerin Therapy: A Case Study and Literature Review | Internal medicine (Tokyo, Japan) |
| *Wadhwani et al. (4)* | 2018 | 2 | Mycotic aortic aneurysms post-Intravesical BCG treatment for early-stage bladder carcinoma | CVIR endovascular |
| *Hui et al. (5)* | 2016 | 1 | Massive Hemoptysis From an Aortobronchial Fistula Secondary to BCG-Related Mycotic Thoracic Aortic Aneurysm | The Annals of thoracic surgery |
| *Coddington et al. (6)* | 2017 | 1 | Mycotic Aneurysm after Bacillus Calmette-Guerin Treatment: Case Report and Review of the Literature | Case reports in urology |
| *Holmes et al. (7)* | 2014 | 1 | Mycotic aortic aneurysm due to intravesical BCG immunotherapy: Clinical manifestations and diagnostic challenges | International journal of mycobacteriology |
| *Floros et al.(8)* | 2015 | 1 | Ruptured Mycotic Aortic Aneurysm after Bacille Calmette-Guerin Therapy | Annals of vascular surgery |
| *Davis et al. (9)* | 2015 | 1 | Successful treatment of a mycotic multifocal thoracoabdominal aortic aneurysm as a late sequelae of intravesical bacillus Calmette-Guerin therapy: case report and literature review | Annals of vascular surgery |
| *Leo et al. (10)* | 2015 | 2 | Mycotic abdominal aortic aneurysm after adjuvant therapy with bacillus Calmette-Guerin in patients with urothelial bladder cancer: a rare but misinterpreted complication | Annals of vascular surgery |
| *Seastedt et al. (11)* | 2015 | 1 | Mycotic Thoracic Aortic Aneurysm After Intravesical Bacillus Calmette-Guerin Treatment | The Annals of thoracic surgery |
| *Akita et al. (12)* | 2015 | 1 | Infectious aortic aneurysms occurring 1 year after bacillus Calmette-Guerin bladder instillation therapy | International journal of urology : official journal of the Japanese Urological Association |
| *Nam et al. (13)* | 2015 | 1 | Infected Aortic Aneurysm caused by Mycobacterium bovis after Intravesical Bacillus Calmette-Guerin Treatment for Bladder Cancer | Infection & chemotherapy |
| *Roylance et al. (14)* | 2013 | 1 | Aorto-enteric fistula development secondary to mycotic abdominal aortic aneurysm following intravesical bacillus Calmette-Guerin (BCG) treatment for transitional cell carcinoma of the bladder | International journal of surgery case reports |
| *Mizoguchi et al. (15)* | 2013 | 1 | Abdominal aortic aneurysmal and endovascular device infection with iliopsoas abscess caused by Mycobacterium bovis as a complication of intravesical bacillus Calmette-Guerin therapy | Annals of vascular surgery |
| *Ventosa-Fernandez et al. (16)* | 2015 | 1 | Infected False Aneurysm of the Aortic Arch After Endoscopic Transurethral Instillation of Bacillus Calmette-Guerin | The Annals of thoracic surgery |
| *Harding et al. (17)* | 2007 | 1 | Ruptured mycotic abdominal aortic aneurysm secondary to Mycobacterium bovis after intravesical treatment with bacillus Calmette-Guerin | Journal of vascular surgery |
| *Santbergen et al. (18)* | 2013 | 1 | Combined infection of vertebroplasty and aortic graft after intravesical BCG treatment | BMJ case reports |
| *Roeke et al. (19)* | 2018 | 1 | A mycotic aneurysm of the abdominal aorta caused by Mycobacterium bovis after intravesical instillation with bacillus Calmette-Guerin | Journal of vascular surgery cases and innovative techniques |
| *Khandelwal et al. (20)* | 2012 | 1 | Ruptured Aortic Aneurysm Secondary to Psoas abscess after Intravesical Bacilli Calmette-Guerin | The Medical journal of Malaysia |
| *Costiniuk et al. (21)* | 2010 | 1 | Mycobacterium bovis abdominal aortic and femoral artery aneurysms following intravesical bacillus Calmette-Guerin therapy for bladder cancer | Cardiovascular pathology : the official journal of the Society for Cardiovascular Pathology |
| *Maundrell et al. (22)* | 2011 | 1 | Mycotic aneurysm of the aorta as a complication of Bacillus Calmette-Guerin instillation | The journal of the Royal College of Physicians of Edinburgh |
| *Wolf et al. (23)* | 1995 | 1 | Infection of a ruptured aortic aneurysm and an aortic graft with bacille Calmette-Guerin after intravesical administration for bladder cancer | Journal of vascular surgery |
| *Lareyre et al. (24)* | 2019 | 1 | Mycotic Aortic Aneurysm and Infected Aortic Graft After Intravesical Bacillus Calmette-Guerin Treatment for Bladder Cancer | Vascular and endovascular surgery |
| *Dahl et al. (25)* | 2005 | 1 | Ruptured abdominal aortic aneurysm secondary to tuberculous spondylitis | International angiology : a journal of the International Union of Angiology |
| *Rozenblit et al. (26)* | 1996 | 1 | Infected aortic aneurysm and vertebral osteomyelitis after intravesical bacillus Calmette-Guerin therapy | AJR. American journal of roentgenology |
| *Yildiz et al. (27)* | 2014 | 1 | An unexpected complication of bacillus Calmette-Guerin therapy | Acta clinica Belgica |
| *LaBerge et al. (28)* | 1999 | 1 | Diagnosis please. Case 9: mycotic pseudoaneurysm of the abdominal aorta in association with mycobacterial psoas abscess--a complication of BCG therapy | Radiology |
| *Psoinos et al. (29)* | 2013 | 1 | A Mycobacterium bovis mycotic abdominal aortic aneurysm resulting from bladder cancer treatment, resection, and reconstruction with a cryopreserved aortic graft | Vascular and endovascular surgery |
| *Woods et al. (30)* | 1988 | 1 | Mycotic abdominal aortic aneurysm induced by immunotherapy with bacille Calmette-Guerin vaccine for malignancy | Journal of vascular surgery |
| *Wada et al. (31)* | 2003 | 1 | Tuberculous abdominal aortic pseudoaneurysm penetrating the left psoas muscle after BCG therapy for bladder cancer | Cardiovascular surgery (London, England) |
| *Darriet et al. (32)* | 2018 | 1 | Fluorescence in situ hybridization microscopic detection of Bacilli Calmette Guerin mycobacteria in aortic lesions: A case report | Medicine (Baltimore) |
| *Pittman et al. (33)* | 2012 | 1 | Primary aortoenteric fistula following disseminated bacillus Calmette-Guerin infection: a case report | Vascular |
| *Hakim et al. (34)* | 1993 | 1 | Psoas abscess following intravesical bacillus Calmette-Guerin for bladder cancer: a case report | The Journal of urology |
| *Damm et al. (35)* | 1997 | 1 | Ruptured mycotic aneurysm of the abdominal aorta: a serious complication of intravesical instillation bacillus Calmette-Guerin therapy | The Journal of urology |
| *Hellinger et al. (36)* | 1995 | 1 | Vascular and other serious infections with Mycobacterium bovis after bacillus of Calmette-Guerin therapy for bladder cancer | Southern medical journal |
| *Kusakabe et al. (37)* | 2018 | 1 | Bacille Calmette-Guerin (BCG) spondylitis with adjacent mycotic aortic aneurysm after intravesical BCG therapy: a case report and literature review | BMC infectious diseases |
| *Samadian et al. (38)* | 2013 | 1 | Mycobacterium bovis vertebral osteomyelitis and discitis with adjacent mycotic abdominal aortic aneurysm caused by intravesical BCG therapy: a case report in an elderly gentleman | Age and ageing |
| *Seelig et al. (39)* | 1999 | 1 | Mycotic vascular infections of large arteries with Mycobacterium bovis after intravesical bacillus Calmette-Guerin therapy: case report | Journal of vascular surgery |
| *Coscas et al. (40)* | 2009 | 1 | Multiple mycotic aneurysms due to Mycobacterium bovis after intravesical bacillus Calmette-Guerin therapy | Journal of vascular surgery |
| *Smith et al. (41)* | 2016 | 1 | BCG-osis following intravesical BCG treatment leading to miliary pulmonary nodules, penile granulomas and a mycotic aortic aneurysm | BMJ case reports |
| *Berchiolli et al. (42)* | 2019 | 1 | Ruptured Mycotic Aneurysm After Intravesical Instillation for Bladder Tumor | Annals of vascular surgery |

1. Okon E, Stearns J, Durgam AK. Tuberculous Psoas Abscess and Worsening Vascular Aneurysm; All from Bacillus Calmette-Guerin (BCG) Therapy? Am J Case Rep. 2017;18:810-2.

2. Simar J, Belkhir L, Tombal B, Andre E. Ruptured aortic aneurysm due to Mycobacterium bovis BCG with a delayed bacteriological diagnosis due to false negative result of the MPB 64 immunochromatographic assay. BMC Res Notes. 2017;10(1):64.

3. Higashi Y, Nakamura S, Kidani K, Matumoto K, Kawago K, Isobe J, et al. Mycobacterium bovis-induced Aneurysm after Intravesical Bacillus Calmette-Guerin Therapy: A Case Study and Literature Review. Intern Med. 2018;57(3):429-35.

4. Wadhwani A, Moore RD, Bakshi D, Mirakhur A. Mycotic aortic aneurysms post-Intravesical BCG treatment for early-stage bladder carcinoma. CVIR Endovasc. 2018;1(1):28.

5. Hui DS, Stoeckel DA, Kaufman EE, Jacobs DL. Massive Hemoptysis From an Aortobronchial Fistula Secondary to BCG-Related Mycotic Thoracic Aortic Aneurysm. Ann Thorac Surg. 2016;101(1):350-2.

6. Coddington ND, Sandberg JK, Yang C, Sehn JK, Kim EH, Strope SA. Mycotic Aneurysm after Bacillus Calmette-Guerin Treatment: Case Report and Review of the Literature. Case Rep Urol. 2017;2017:4508583.

7. Holmes BJ, LaRue RW, Black JH, 3rd, Dionne K, Parrish NM, Melia MT. Mycotic aortic aneurysm due to intravesical BCG immunotherapy: Clinical manifestations and diagnostic challenges. Int J Mycobacteriol. 2014;3(1):60-5.

8. Floros N, Meletiadis K, Kusenack U, Zirngibl H, Kamper L, Haage P, et al. Ruptured Mycotic Aortic Aneurysm after Bacille Calmette-Guerin Therapy. Ann Vasc Surg. 2015;29(7):1450.e1-4.

9. Davis FM, Miller DJ, Newton D, Arya S, Escobar GA. Successful treatment of a mycotic multifocal thoracoabdominal aortic aneurysm as a late sequelae of intravesical bacillus Calmette-Guerin therapy: case report and literature review. Ann Vasc Surg. 2015;29(4):840.e9-13.

10. Leo E, Molinari AL, Rossi G, Ferrari SA, Terzi A, Lorenzi G. Mycotic abdominal aortic aneurysm after adjuvant therapy with bacillus Calmette-Guerin in patients with urothelial bladder cancer: a rare but misinterpreted complication. Ann Vasc Surg. 2015;29(6):1318.e1-6.

11. Seastedt KP, Ahmad U, Lau C, Ruggeri-Weigel P, Tsang HC, Hartman BJ, et al. Mycotic Thoracic Aortic Aneurysm After Intravesical Bacillus Calmette-Guerin Treatment. Ann Thorac Surg. 2015;99(6):2210-2.

12. Akita H, Okamura T, Nakane A, Kobayashi T, Yamada K, Tanaka Y. Infectious aortic aneurysms occurring 1 year after bacillus Calmette-Guerin bladder instillation therapy. Int J Urol. 2015;22(2):234-5.

13. Nam EY, Na SH, Kim SY, Yoon D, Kim CJ, Park KU, et al. Infected Aortic Aneurysm caused by Mycobacterium bovis after Intravesical Bacillus Calmette-Guerin Treatment for Bladder Cancer. Infect Chemother. 2015;47(4):256-60.

14. Roylance A, Mosley J, Jameel M, Sylvan A, Walker V. Aorto-enteric fistula development secondary to mycotic abdominal aortic aneurysm following intravesical bacillus Calmette-Guerin (BCG) treatment for transitional cell carcinoma of the bladder. Int J Surg Case Rep. 2013;4(1):88-90.

15. Mizoguchi H, Iida O, Dohi T, Tomoda K, Kimura H, Inoue K, et al. Abdominal aortic aneurysmal and endovascular device infection with iliopsoas abscess caused by Mycobacterium bovis as a complication of intravesical bacillus Calmette-Guerin therapy. Ann Vasc Surg. 2013;27(8):1186.e1-5.

16. Ventosa-Fernandez G, Milisenda JC, Pereda D, Mestres CA. Infected False Aneurysm of the Aortic Arch After Endoscopic Transurethral Instillation of Bacillus Calmette-Guerin. Ann Thorac Surg. 2015;100(2):717-20.

17. Harding GE, Lawlor DK. Ruptured mycotic abdominal aortic aneurysm secondary to Mycobacterium bovis after intravesical treatment with bacillus Calmette-Guerin. J Vasc Surg. 2007;46(1):131-4.

18. Santbergen B, Vriens PH, de Lange WC, Van Kasteren ME. Combined infection of vertebroplasty and aortic graft after intravesical BCG treatment. BMJ Case Rep. 2013;2013.

19. Roeke T, Hovsibian S, Schlejen PM, Dinant S, Koster T, Waasdorp EJ. A mycotic aneurysm of the abdominal aorta caused by Mycobacterium bovis after intravesical instillation with bacillus Calmette-Guerin. J Vasc Surg Cases Innov Tech. 2018;4(2):122-5.

20. Khandelwal A, Gupta A, Virmani V, Khandelwal K. Ruptured Aortic Aneurysm Secondary to Psoas abscess after Intravesical Bacilli Calmette-Guerin. Med J Malaysia. 2012;67(5):534-5.

21. Costiniuk CT, Sharapov AA, Rose GW, Veinot JP, Desjardins M, Brandys TM, et al. Mycobacterium bovis abdominal aortic and femoral artery aneurysms following intravesical bacillus Calmette-Guerin therapy for bladder cancer. Cardiovasc Pathol. 2010;19(2):e29-32.

22. Maundrell J, Fletcher S, Roberts P, Stein A, Lambie M. Mycotic aneurysm of the aorta as a complication of Bacillus Calmette-Guerin instillation. J R Coll Physicians Edinb. 2011;41(2):114-6.

23. Wolf YG, Wolf DG, Higginbottom PA, Dilley RB. Infection of a ruptured aortic aneurysm and an aortic graft with bacille Calmette-Guerin after intravesical administration for bladder cancer. J Vasc Surg. 1995;22(1):80-4.

24. Lareyre F, Reverso-Meinietti J, Carboni J, Gaudart A, Hassen-Khodja R, Raffort JM. Mycotic Aortic Aneurysm and Infected Aortic Graft After Intravesical Bacillus Calmette-Guerin Treatment for Bladder Cancer. Vasc Endovascular Surg. 2019;53(1):86-91.

25. Dahl T, Lange C, Odegard A, Bergh K, Osen SS, Myhre HO. Ruptured abdominal aortic aneurysm secondary to tuberculous spondylitis. Int Angiol. 2005;24(1):98-101.

26. Rozenblit A, Wasserman E, Marin ML, Veith FJ, Cynamon J, Rozenblit G. Infected aortic aneurysm and vertebral osteomyelitis after intravesical bacillus Calmette-Guerin therapy. AJR Am J Roentgenol. 1996;167(3):711-3.

27. Yildiz H, Colin G, Lambert M. An unexpected complication of bacillus Calmette-Guerin therapy. Acta Clin Belg. 2014;69(4):312.

28. LaBerge JM, Kerlan RK, Jr., Reilly LM, Chuter TA. Diagnosis please. Case 9: mycotic pseudoaneurysm of the abdominal aorta in association with mycobacterial psoas abscess--a complication of BCG therapy. Radiology. 1999;211(1):81-5.

29. Psoinos CM, Simons JP, Baril DT, Robinson WP, Schanzer A. A Mycobacterium bovis mycotic abdominal aortic aneurysm resulting from bladder cancer treatment, resection, and reconstruction with a cryopreserved aortic graft. Vasc Endovascular Surg. 2013;47(1):61-4.

30. Woods JMt, Schellack J, Stewart MT, Murray DR, Schwartzman SW. Mycotic abdominal aortic aneurysm induced by immunotherapy with bacille Calmette-Guerin vaccine for malignancy. J Vasc Surg. 1988;7(6):808-10.

31. Wada S, Watanabe Y, Shiono N, Masuhara H, Hamada S, Ozawa T, et al. Tuberculous abdominal aortic pseudoaneurysm penetrating the left psoas muscle after BCG therapy for bladder cancer. Cardiovasc Surg. 2003;11(3):231-5.

32. Darriet F, Bernioles P, Loukil A, Saidani N, Eldin C, Drancourt M. Fluorescence in situ hybridization microscopic detection of Bacilli Calmette Guerin mycobacteria in aortic lesions: A case report. Medicine (Baltimore). 2018;97(30):e11321.

33. Pittman M, Sakai L, Craig R, Joehl R, Milner R. Primary aortoenteric fistula following disseminated bacillus Calmette-Guerin infection: a case report. Vascular. 2012;20(4):221-4.

34. Hakim S, Heaney JA, Heinz T, Zwolak RW. Psoas abscess following intravesical bacillus Calmette-Guerin for bladder cancer: a case report. J Urol. 1993;150(1):188-9.

35. Damm O, Briheim G, Hagstrom T, Jonsson B, Skau T. Ruptured mycotic aneurysm of the abdominal aorta: a serious complication of intravesical instillation bacillus Calmette-Guerin therapy. J Urol. 1998;159(3):984.

36. Hellinger WC, Oldenburg WA, Alvarez S. Vascular and other serious infections with Mycobacterium bovis after bacillus of Calmette-Guerin therapy for bladder cancer. South Med J. 1995;88(12):1212-6.

37. Kusakabe T, Endo K, Nakamura I, Suzuki H, Nishimura H, Fukushima S, et al. Bacille Calmette-Guerin (BCG) spondylitis with adjacent mycotic aortic aneurysm after intravesical BCG therapy: a case report and literature review. BMC Infect Dis. 2018;18(1):290.

38. Samadian S, Phillips FM, Deeab D. Mycobacterium bovis vertebral osteomyelitis and discitis with adjacent mycotic abdominal aortic aneurysm caused by intravesical BCG therapy: a case report in an elderly gentleman. Age Ageing. 2013;42(1):129-31.

39. Seelig MH, Oldenburg WA, Klingler PJ, Blute ML, Pairolero PC. Mycotic vascular infections of large arteries with Mycobacterium bovis after intravesical bacillus Calmette-Guerin therapy: case report. J Vasc Surg. 1999;29(2):377-81.

40. Coscas R, Arlet JB, Belhomme D, Fabiani JN, Pouchot J. Multiple mycotic aneurysms due to Mycobacterium bovis after intravesical bacillus Calmette-Guerin therapy. J Vasc Surg. 2009;50(5):1185-90.

41. Smith DM. BCG-osis following intravesical BCG treatment leading to miliary pulmonary nodules, penile granulomas and a mycotic aortic aneurysm. BMJ Case Rep. 2016;2016.

42. Berchiolli R, Mocellin DM, Marconi M, Tomei F, Bargellini I, Zanca R, et al. Ruptured Mycotic Aneurysm After Intravesical Instillation for Bladder Tumor. Ann Vasc Surg. 2019;59:310.e7-.e11.
