# Supplementary figures and images for: Aortic aneurysm and aortic graft infection related to Mycobacterium bovis after intravesical Bacille Calmette–Guérin therapy—a case series
Source: BMC Surg. 2021 Mar 17;21:138. doi: 10.1186/s12893-021-01142-1 (PMC7972206; doi:10.1186/s12893-021-01142-1)

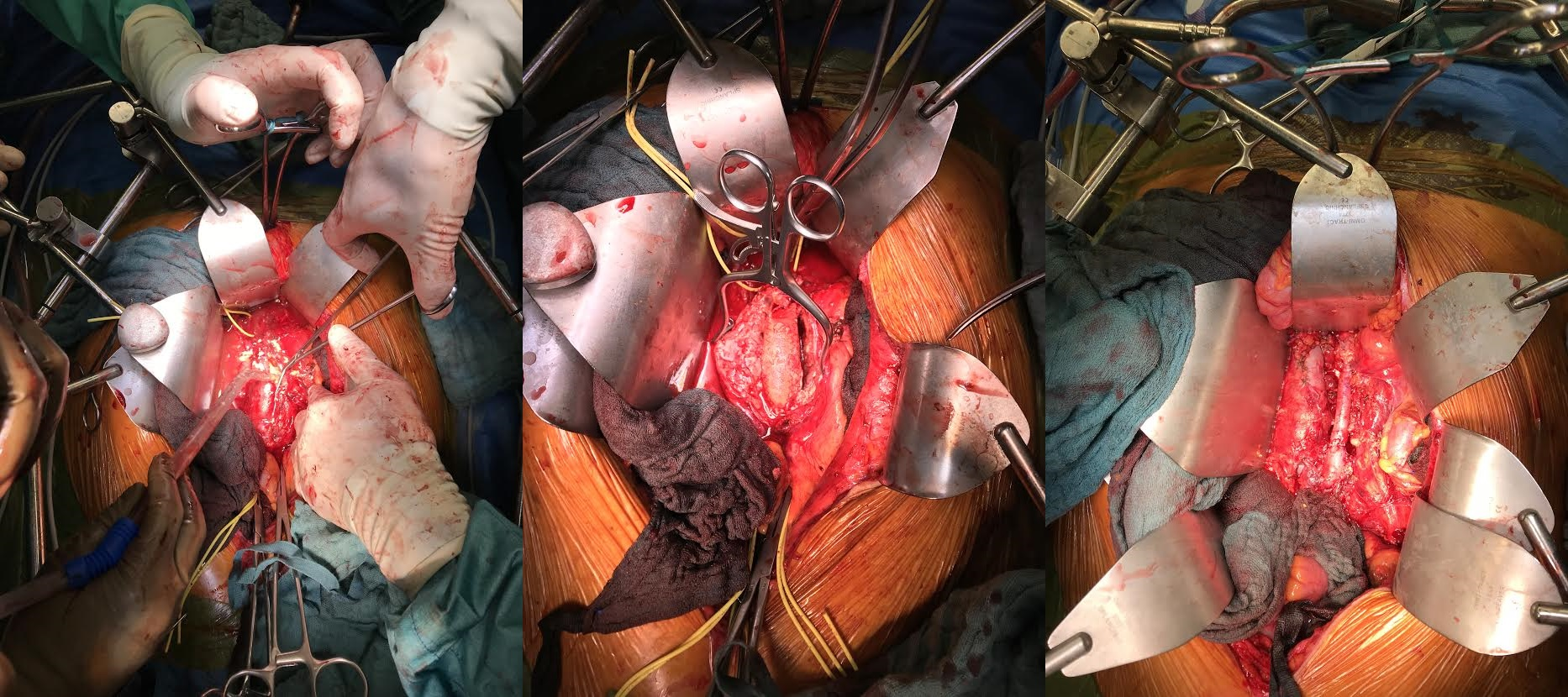

Supplement: Supplementary file 2 — Additional file 2: Fig. S1. Intraoperative situs showing exit of purulent granular material from the aneurysmatic formation (A) surrounding former implanted Dacron tube graft (B) and aortic reconstruction after graft removal and reconstruction with deep femoral vein (C) [file 12893_2021_1142_MOESM2_ESM.tif]
